# Health and well-being of children and adolescents living in the Kimberley region of Western Australia: a scoping review protocol

**DOI:** 10.1136/bmjopen-2024-098268

**Published:** 2025-08-06

**Authors:** Lisa Cannon, Emily Carter, Jadnah Davies, Sue Thomas, Elizabeth J Elliott, Lauren J Rice

**Affiliations:** 1 Faculty of Medicine and Health, Speciality of Child and Adolescent Health, The University of Sydney, Sydney, New South Wales, Australia; 2 Marulu Team, Marninwarntikura Women’s Resource Centre, Fitzroy Crossing, Western Australia, Australia; 3 Sydney Children’s Hospitals Network, Kids Research, Sydney, New South Wales, Australia

**Keywords:** Australian Aboriginal and Torres Strait Islander Peoples, Child, Adolescent, Health, MENTAL HEALTH

## Abstract

**Abstract:**

**Introduction:**

Aboriginal people in the Kimberley are concerned that scientific research, government Inquiries and Royal Commissions are not adequately informing policy and service design. In this protocol paper, we outline our proposed scoping review to identify and provide a broad overview of scientific literature regarding the health, well-being, mental health, disability, education and social outcomes of children and adolescents living in the Kimberley region of Western Australia and the recommendations that came from them.

**Methods and analysis:**

This scoping review is guided by Arksey and O’Malley’s (2005) methodological framework. We will conduct a comprehensive search across multiple databases using several search engines. Inclusion criteria were established to inform the selection of papers to be included in the review. After de-duplication, all titles and abstracts will be reviewed, followed by full-text screening. A second reviewer will independently screen 20% of the titles, abstracts and full texts. Two reviewers will discuss discrepancies, and a third reviewer will resolve any disagreements that may arise. We will use a data extraction template in Covidence to systematically extract relevant data.

**Ethics and dissemination:**

This scoping review does not require ethics approval, as we are investigating the breadth of existing literature regarding the outcomes of children and adolescents in the Kimberley, Western Australia. The scoping review results will be published in peer-reviewed journal(s) and shared with relevant policymakers to help inform future policies and service improvements and designs in the region.

**STRENGTHS AND LIMITATIONS OF THIS STUDY**

- This scoping review collates journal articles on the health, mental health, educational and social outcomes of children and adolescents in the Kimberley region of Western Australia.
- The review follows an established scoping review framework methodology and is reported in accordance with the Preferred Reporting Items for Systematic Reviews and Meta-Analyses extension for Scoping Reviews.
- As the review focuses on the Kimberley, the findings may not be generalisable to other regions.
- Grey literature, such as government or community reports, is beyond the scope of this review.

## Introduction

 The Kimberley is the northernmost region of Western Australia, spanning 423 000 km^2^. It contains outstanding natural features, ancient and protected seas, and landscapes of great cultural significance.^1^ Aboriginal people have resided in the Kimberley for at least 50 000 years^2 3^ and have worked hard to protect their people, country and culture following the European invasion.^4^,^7^ The Kimberley includes over 150 Aboriginal communities and 6 towns.^8^ Aboriginal people represent 41% of the Kimberley population, compared with 3.3% of the Western Australian population (Aboriginal people represent 60.2% of the population in West Kimberley).^9 10^ The high proportion of Aboriginal people living in the area, along with their efforts to protect their land and culture, has meant that the region remains prosperous with a traditional Aboriginal way of life. Many Aboriginal people continue to use their traditional languages, preserve sophisticated kinship systems, and live on or near their ancestral lands, practicing their cultural traditions and customs.

Despite the many strengths of Aboriginal people in the Kimberley, they, like most Aboriginal and Torres Strait Islander people, continue to experience the adverse effects of colonisation, including significant health^11^ and socioeconomic inequities.^12^ The Kimberley has been extensively researched in an attempt to address some of this disparity.^8^ There have also been many government Inquiries and Royal Commissions that have included Aboriginal people from the region.^13^,^18^ Research and government-led Inquiries should serve as opportunities for Aboriginal people to identify key issues and solutions required to protect their way of life. This information can then be collaboratively used with government authorities to create meaningful change. However, EC, an Aboriginal author and leader in the Kimberley, is concerned that the research findings and recommendations from Inquiries and Commissions do not always inform government policy and service design or lead to significant change for her community. This concern stems from over 20 years of experience advocating for and consulting with government officials on community issues, particularly those affecting women, children and young people.

Other Aboriginal people across the Kimberley share EC’s concerns. Researchers from the Kimberley Aboriginal Health Research Alliance reviewed the characteristics and outcomes of Aboriginal health research in the Kimberley from 2006 to 2020. They found that translational research benefits for the community were not always evident. As part of this study, the team conducted interviews with 15 Kimberley-based individuals involved in the research, including 11 Aboriginal participants. The interviewees were concerned that research findings were not sufficiently translated into improved services.^8^

Together with others, authors EC and EE have led a 16-year partnership between Marninwarntikura Women’s Resource Centre, an Aboriginal Community Controlled Organisation (ACCO), and the University of Sydney. The partnership was established at the request of senior Aboriginal women from the Fitzroy Valley, in the central west of the Kimberley, to help identify and address the concerns of their Elders about the needs of their grandchildren. The partnership successfully identified key issues facing children and young people in the Fitzroy Valley and raised awareness internationally, including at the United Nations. The partnership also resulted in government investment in ACCO services, including establishing an early childhood centre, a programme to support families with children with disabilities, a parenting programme, and a young adult social and emotional well-being service. For details of the work conducted under this partnership, see Pickard *et al*.^19^ However, more systemic change, particularly within government services, is needed to ensure children in the Kimberley have access to the support and services they need to thrive.

This need is reflected in the National Agreement on Closing the Gap (CTG), developed by the Australian government in consultation with Aboriginal and Torres Strait Islander people.^20^ Eight of the 17 CTG targets relate to improving child outcomes. Nationally, after eight years, only two of these targets are on track (target 2: babies are born healthy and strong and target 3: children are engaged in high-quality, culturally appropriate early childhood education in their early years). For the remaining six targets, improvement has been seen in two (target 5: students achieve their full learning potential and target 7: youth are engaged in education or employment), one is unchanged (target 11: young people are not over-represented in the criminal justice system) and two have worsened (target 4: children thrive in their early years and target 12: children are not over-represented in the child protection system).^20^

The CTG agreement was developed around four priority reforms designed to inform and measure changes in how governments work with Aboriginal and Torres Strait Islander people. The reforms focus on genuine partnerships and shared decision-making, strengthening ACCOs, transforming government organisations and sharing access to data and information at the regional level. The reforms also push for place-based policymaking.^20^ A place-based approach is broadly described as ‘adapt operations to suit a local situation and collaborate with other agencies operating there, and to coordinate the efforts of multiple government silos and tiers’ p.147.^21^ An advantage of this approach is that it focuses on the complexity of issues and attempts to address them comprehensively at all levels. The place-based approach is also considered to align better with traditional Aboriginal governance models than other models of policy design and implementation.^21^

To address concerns about the extent to which research informs government policy in the Kimberley, we designed a scoping review to identify and summarise published scientific literature regarding the health, mental health, disability, education and social outcomes of children and adolescents living in the Kimberley region of Western Australia. We are not aware of any prior reviews being conducted on this topic. A broad summation of the existing literature will identify what is known about the outcomes of children in the Kimberley and what recommendations authors have made for translating these findings into change. Policymakers and advocates can use this summary to inform future place-based service design. The findings will also identify potential gaps in knowledge, ensuring that new research builds on the existing literature.

## Methods

### Protocol design

Given our aim is to provide a broad overview of the literature, we have chosen a scoping review rather than a systematic review.^22 23^ This scoping review follows the five-stage methodology outlined in Arksey and O’Malley’s framework for scoping reviews^22^ and is also informed by more recent recommendations.^24^,^26^ The review will be reported according to the Preferred Reporting Items for Systematic Reviews and Meta-Analyses extension for Scoping Reviews (PRISMA-ScR).^27^

### Stage 1: identifying the research question

The scoping review aims to provide a broad overview of the health, mental health, disability, education and social outcomes of children and adolescents (aged 18 years and under) in the remote Kimberley region of Western Australia. Specifically, we want to understand (a) what topics have been researched; (b) what these publications tell us about the outcomes of children and adolescents; (c) whether research was led by Aboriginal organisations in the Kimberley and (d) what recommendations are provided by the authors that will impact clinical and public health practice and policy.

### Stage 2: identifying relevant studies

#### Eligibility criteria

The eligibility criteria for this scoping review will allow us to capture a wide range of literature regarding children and adolescents in the Kimberley. There are no limits on the type of analyses or study design. Articles presenting qualitative data, quantitative data and meta-analyses, as well as literature reviews and theoretical and conceptual articles, will be included. Papers must be accessible online, peer-reviewed, published in academic journals since 1967, in the English language, and be attentive to the health, mental health, disability, educational and/or social outcomes of children and/or adolescents (aged ≤18 years) in the Kimberley (table 1). The date of 1967 was chosen to align with the 1967 referendum, in which 90.77% of Australians voted to amend the Constitution to remove discriminatory references to Aboriginal and Torres Strait Islander peoples and enable them to be counted as part of the Australian population.

**Table 1:** Eligibility criteria

Availability	Online accessibility
Language	English
Publication Date	January 1967—date of data extraction
Topic/Focus	Health, mental health, disability, education and social outcomes of children and adolescents
Population	Aboriginal and non-Aboriginal children and adolescents aged 18 years and under. Eligible papers can be exclusively about this age group or provide analysis/information about this age group. Papers in which participants are over 18 years of age can be included if the research itself is focused on children and adolescents.
Location/Setting	Kimberley, Western Australia This includes research conducted exclusively within the region, research that includes a sub-analysis/information on participants from the region or research in which most participants come from the region.
Publication Type	Peer-reviewed
Type of Study	No restrictions

#### Information sources

Box 1 shows the search interfaces used and databases that will be systematically searched.

Box 1 **Information Sources**

**Search interface/database**

**Ovid:**

- Joanna Briggs Institute Evidence Based Practice (JBI EBP) Database; all Evidence-Based Medicine Reviews (EBMR); Embase Classic + Embase; Global Health; Ovid Medline; PsycInfo

**PubMed Central**

**ProQuest Central:**

- Public Health Database

**Web of Science**

**EBSCO:**

- MEDLINE, Education Source, SocINDEX with Full Text, Health Source: Nursing/Academic Edition, Psychology and Behavioural Sciences Collection, Cumulative Index to Nursing and Allied Health Literature (CINAHL) Plus, Humanities International Complete, Health Source—Consumer Edition

**AUSThealth:**

- Australasian Medical Index, Australian Public Affairs Information Service (APAIS) Health, ATSI Health, AusportMed, Cinch-Health, DRUG database, HIVA, Health & Society and Rural and Remote Health database

**Table 2:** Concepts and search terms

Concepts	Search terms
Topic—health, mental health, disability, education and social outcomes	‘health*’, ‘mental health*’, ‘disability’, ‘disabilities’, ‘well-being’, ‘depression*’, ‘depressed’, ‘suicide*’, ‘suicidal*’, ‘sexual health’, ‘anxiety’, ‘PTSD’, ‘post-traumatic stress disorder’, ‘quality of life’, ‘wellness’, ‘FASD’, ‘f#etal alcohol spectrum disorder*’, ‘f#etal alcohol syndrome’, ‘FAS’, ‘social determinants of health’, ‘education*’, ‘social outcome*’, ‘health service*’, ‘justice*’, ‘police’, ‘criminal*’, ‘crime*’, ‘child protection’, ‘welfare*’, ‘mental health service*’, ‘youth service*’
Population—children and adolescents	‘Child’, ‘Children’, ‘Adolescent’, ‘Adolescents’, ‘Adolescence’, ‘Youth’, ‘Youths’, ‘Teenage*’
Location—Kimberley, Western Australia	‘Kimberley’, ‘Bilingurr’, ‘Broome’, ‘Cable Beach’, ‘Camballin’, ‘Dampier Peninsula’, ‘Derby’, ‘Derby-West Kimberley’, ‘Djugun’, ‘Eighty Mile Beach’, ‘Fitzroy Crossing’, ‘Geegully Creek’, ‘Gingerah’, ‘Hall Point’, ‘Halls Creek’, ‘Kimbolton’, ‘King Leopold Ranges’, ‘Koolan’, ‘Kununurra’, ‘Lagrange’, ‘Mcbeath’, ‘Meda’, ‘Minyirr’, ‘Mount Hardman’, ‘Roebuck’, ‘St George Ranges’, ‘Turkey Creek’, ‘Warmun’, ‘Waterbank’, ‘Willard’, ‘Wyndham’, ‘Wyndham-East Kimberley’

Where appropriate, truncation symbols (*) and various spelling was included (e.g. foetal and fetal; wellbeing and well-being). The Boolean operator ‘OR’ was included between each search term, and ‘AND’ was used to connect each concept.

#### Search strategy

Several databases were initially searched to identify keywords and potential search terms. A preliminary search of grey literature, including government websites, was also conducted. Due to the large volume of literature identified in the scientific and grey literature sources, it was decided that this review would focus only on the scientific literature. The preliminary search of databases informed the final search query for each database using terms related to the topic, location and population of interest. To ensure a comprehensive scope of the literature, we intentionally included broad terms such as ‘health’ and ‘mental health’, as well as specific terms like ‘fetal alcohol spectrum disorder’. Given our familiarity with the region and the existing literature, we anticipate that these terms will yield valuable insights. The specific search terms used are provided in table 2, and the search strategy for PubMed Central, including limits, is provided in online supplemental material 1. We consulted with a librarian when formulating the list of search interfaces/databases described in box 1 and the search terms listed in table 2. The search will be completed by the end of 2025.

### Stage 3: study selection

Papers will be imported into EndNote, and all duplicates will be removed by the co-first author (LC) before title/abstract screening is undertaken. Papers will be divided among at least two researchers who will conduct title/abstract screening independently. The researchers will be guided by the eligibility criteria outlined in table 1 and encouraged to reach out to LC for further clarification if needed. To reduce the premature exclusion of potentially relevant papers, researchers will have the option of sorting papers into ‘relevant’, ‘not relevant’ and ‘unsure’ folders. Papers that are sorted into the ‘unsure’ folder will be reviewed by two researchers who will meet and discuss.

Papers sorted into ‘relevant’ and ‘unsure’ folders will move onto the full-text screening stage. Using Covidence, full texts will be screened for eligibility by LC, and another researcher will initially screen a random sample of 20% of these papers. Any discrepancies will be discussed and resolved by the two researchers. If a consensus cannot be reached, a third researcher will make the final decision. If there is a high level of agreement (≥90%) between LC and the second researcher, the second researcher will only conduct full-text screening on publications where there is some uncertainty in eligibility. In the event of a significant number of discrepancies, the two researchers will convene to review the eligibility criteria and, if necessary, provide further clarification. Following this, the second researcher will screen an additional 20% of publications before reconvening to discuss any discrepancies. This process will continue until a high level of agreement is reached. LC will search the reference lists of all relevant papers to identify any potentially relevant papers. These papers will then undergo the screening process. The study selection process will be reported in the review using the Preferred Reporting Items for Systematic Reviews and Meta-Analyses (PRISMA) flow diagram.

### Stage 4: data extraction (charting the data)

Information from all relevant papers will be extracted using a customised data extraction template developed for this scoping review. The template (table 3), with detailed instructions, will be added to the data extraction template in Covidence. Papers will be sorted into broad categories, each assigned to a researcher to conduct data extraction in Covidence independently. Researchers involved in data extraction will meet with the co-first author (LC) to discuss the template, the information to be extracted and the broad level of detail required. Additionally, the template will include a designated open text field for researchers to provide additional notes regarding the data extraction process. For example, this section may be used to recommend a second researcher review the paper or request verification of information provided under a particular field. The focus of this review is to identify broad research outcome topics and recommendations rather than examine the quality of specific studies. We therefore will not conduct risk of bias assessments.

**Table 3:** Finalised data extraction template in Covidence

Items	Details
Basic descriptive data	Author(s) (open text) Title of the Paper (open text) Year of Publication (open text) APA reference (open text)
Author affiliation	Were any of the authors’ affiliations associated with/located within the Kimberley? (yes/no). If yes, how many authors, and what were the affiliations (open text)?
Collaboration/engagement with the community	Did the paper mention any consultation, collaboration and/or engagement with the community (including information provided in the acknowledgements section) (yes/no)? If yes, briefly describe (open text).
Aim of the study	Summarise the aim of the study (open text).
Location	List the locations included in the study that are within the Kimberley. If available, also include the setting (eg, school, hospital) (open text). List the locations included in the study that are outside the Kimberley (open text).
Study design	Select (single choice): randomised controlled trial; Non-randomised experimental study; cohort study; cross-sectional study; case-control study; systematic review; qualitative research; prevalence study; case series; case report; diagnostic test accuracy study; clinical prediction rule; economic evaluation; text and opinion; other (specify; open text)
Data and measures	List/briefly describe the type of data, measurements and/or assessment tools (open text).
Participants/population	Provide the total N and age range/s for the overall study (open text). Provide characteristics specifically regarding participants aged 0–18 years old within the Kimberley (e.g., n; age range/age groups, gender and other characteristics available) (open text).
Main results	Summarise the main findings, focusing on those related to people aged 0–18 years old in the Kimberley. If available, also include comparison data that may be of interest (open text).
Outcomes of young people in the Kimberley	What do these publications tell us about the outcomes of young people in the Kimberley? For example, prevalence, incidence and comparison between/within different communities (open text).
Risk and protective factors	Provide (open text) the attributes, characteristics or exposures that are mentioned in the paper (and the reasoning provided by the authors) that could: Influence the young people’s outcomes or increase their likelihood of developing the disease or health issue. Have a positive influence on the young people’s outcomes or decrease their likelihood of developing the disease or health issue.
Recommendations made by the authors	Provide details explaining what (if any) recommendations were made by the authors to address these outcomes and why these recommendations were made (open text).
Concluding remarks	Provide any additional information that is of importance (optional; open text).
ICD-11 Coding Tool*	For papers that focus on health, use the ICD-11 Coding Tool (https://icd.who.int/en) to state what ICD-11 chapter (ie, first-level item) and block (ie, second-level item) the primary disease/disorder falls under.
Additional notes (optional)	If you have any comments related to the review and/or data you have extracted above, for example, 'need a second opinion'.

* ICD-11 Coding Tool: International Classification of Diseases (11th Revision) for Mortality and Morbidity Statistics (ICD-11 MMS).

### Stage 5: collating, summarising and reporting results

Data analysis in scoping reviews is generally characterised by a descriptive approach that focuses on basic frequencies and percentages.^26^ Descriptive data, such as year of publication and location, will be reported in a table where relevant. Papers will be divided into themes, with the ICD-11 coding guiding the themes and subthemes for health-related papers. To provide further understanding of the literature, additional information in relation to the review objectives will be reported for each theme. This will include a broad overview of what topic areas have been researched and what these publications tell us about the outcomes of children and adolescents. The method of reporting risk factors, protective factors and the recommendations provided by the authors will depend on the contents of the extracted information. Data extraction is expected to be completed by the end of 2025.

## Patient and public involvement

This study was conducted as a part of a 16-year partnership between senior Aboriginal women from the Kimberley, currently represented by Marninwarntikura Women’s Resource Centre (an ACCO in Fitzroy Valley, in the central west of the Kimberley, Western Australia), and researchers and clinicians from the University of Sydney, New South Wales. The partnership was established at the request of senior Aboriginal women from the Fitzroy Valley to help identify and address the concerns of community leaders about the needs of their grandchildren. Four of the six authors of this paper are researchers living in the Kimberley (EC, JD, ST and LJR). EC is a Gooniyandi and Kija woman and CEO of the Marninwarntikura Women’s Resource Centre, while JD is a Gooniyandi, Kija and Waanyi woman. We used reflexive practices to consider how our life experiences shape our perspectives during the research and writing of this paper.

## Ethics and dissemination

This is the first scoping review of the health, mental health, disability, educational and social outcomes of children and adolescents living in the Kimberley region of Western Australia. The review will identify recommendations arising from the included papers and help in informing place-based policy and service improvement/design. The findings will also identify potential gaps in knowledge, ensuring that new research builds upon the existing literature. The results will be disseminated through peer-reviewed journals and policy briefs for government departments, ACCOs and other key stakeholders. This review is restricted to articles published in scientific journals and excludes grey literature, such as local or government reports. Ethical approval is not required for the scoping review.

## Supplementary material

10.1136/bmjopen-2024-098268 online supplemental file 1

## References

[1] Cvitanovic C, Mackay M, Kelly R (2021). Thirty critical research needs for managing an ecologically and culturally unique remote marine environment: The Kimberley region of Western Australia. Ocean Coast Manag.

[2] Clarkson C, Norman K, O’Connor S, McNiven IJ, David B (2021). The Oxford handbook of the archaeology of Indigenous Australia and New Guinea.

[3] Norman K, Shipton C, O’Connor S (2022). Human occupation of the Kimberley coast of northwest Australia 50,000 years ago. Quat Sci Rev.

[4] Dudgeon P, Wright M, Paradies Y, Dudgeon P, Milroy H, Walker R (2010). Working together: Aboriginal and Torres Strait Islander mental health and wellbeing principles and practice.

[5] Filev A, Pedersen J, Oscar J (2022). Wiyi yani u thangani (women’s voices): first nations women’s safety policy forum outcomes report - November 2022.

[6] Lindsay M, Beames L, Yawuru Country Managers (2022). Integrating scientific and Aboriginal knowledge, practice and priorities to conserve an endangered rainforest ecosystem in the Kimberley region, northern Australia. Eco Management Restoration.

[7] Woorunmurra B, Pedersen H (2011). Jandamarra and the Bunuba resistance.

[8] Matsumoto A, Blackburn K, Spicer B (2023). A Mixed Methods Study of 15 Years of Aboriginal Health Research in the Kimberley: 'We’ve Been Researched, We Think, from Head to Toe, Inside and Outside, Upside Down'. Int J Environ Res Public Health.

[9] Australian Bureau of Statistics (2021). Region summary: Kimberley. https://dbr.abs.gov.au/region.html?lyr=sa3&rgn=51001.

[10] Australian Bureau of Statistics (2021). Region summary: Western Australia. https://dbr.abs.gov.au/region.html?lyr=ste&rgn=5.

[11] Government of Western Australia (2022). Kimberley health profile.

[12] Australian Bureau of Statistics (2021). Socio-economic indexes for Australia local government area: Commonwealth of Australia. https://www.abs.gov.au/statistics/people/people-and-communities/socio-economic-indexes-areas-seifa-australia/2021/Local%20Government%20Area%2C%20Indexes%2C%20SEIFA%202021.xlsx.

[13] Commonwealth of Australia (1991). Royal Commission into Aboriginal deaths in custody - national report. contract no.: 130.

[14] Fogliani RVC (2019). Inquest into the 13 deaths of children and young persons in the Kimberley region: inquest finding.

[15] Hope AN (2008). Inquest into Indigenous suicides in the Kimberley.

[16] Senate Community Affairs References Committee (2021). Effective approaches to prevention, diagnosis and support for fetal alcohol spectrum disorder.

[17] Western Australian Legislative Assembly Education and Health Standing Committee (2012). Fetal alcohol spectrum disorder: the invisible disability. Report 15.

[18] (2023). The Royal Commission into violence, abuse, neglect and exploitation of people with disability. First nations people with disability final report.

[19] Pickard A, Stubbs T, Carter E (2025). Aboriginal Community Controlled Organisations Leading the Way in Child Health Research. J Community Health.

[20] Commonwealth of Australia, Department of the Prime Minister and Cabinet (2020). Closing the gap report 2020 (1925364291).

[21] Hart A, Connolly J (2022). Commonwealth place‐based policies for addressing geographically concentrated disadvantage: A typology and critical analysis. Aust J Public Adm.

[22] Arksey H, O’Malley L (2005). Scoping studies: towards a methodological framework. Int J Soc Res Methodol.

[23] Munn Z, Peters MDJ, Stern C (2018). Systematic review or scoping review? Guidance for authors when choosing between a systematic or scoping review approach. BMC Med Res Methodol.

[24] Levac D, Colquhoun H, O’Brien KK (2010). Scoping studies: advancing the methodology. Implementation Sci.

[25] Peters MDJ, Godfrey CM, Khalil H (2015). Guidance for conducting systematic scoping reviews. Int J Evid Based Healthc.

[26] Peters MDJ, Godfrey C, McInerney P (2022). Best practice guidance and reporting items for the development of scoping review protocols. JBI Evid Synth.

[27] Tricco AC, Lillie E, Zarin W (2018). PRISMA Extension for Scoping Reviews (PRISMA-ScR): Checklist and Explanation. Ann Intern Med.

