# Supplementary material for: Health and well-being of children and adolescents living in the Kimberley region of Western Australia: a scoping review protocol
Source: BMJ Open. 2025 Aug 6;15(8):e098268. doi: 10.1136/bmjopen-2024-098268 (PMC12336523; doi:10.1136/bmjopen-2024-098268)
Supplement: online supplemental file 1 [file bmjopen-15-8-s001.pdf]

# Health and well-being of children and adolescents living in the Kimberley region of Western Australia: a scoping review protocol

## Supplementary Material 1: Search Strategy Template

| Platform/Database: (eg PubMed Central) | | |
|---|---|---|
| Date of Search: [dd/mm/yy] | | |
| ID | Terms | Results |
| 1 | (("health*") OR ("mental health*") OR ("disability") OR ("disabilities") OR ("wellbeing") OR ("well being") OR ("well-being") OR ("depression*") OR ("depressed") OR ("suicide*") OR ("suicidal*") OR ("sexual health") OR ("anxiety") OR ("PTSD") OR ("post-traumatic stress disorder") OR ("quality of life") OR ("wellness") OR ("FASD") OR ("fetal alcohol spectrum disorder") OR ("fetal alcohol spectrum disorders") OR ("foetal alcohol spectrum disorder") OR ("foetal alcohol spectrum disorders") OR ("fetal alcohol syndrome") OR ("foetal alcohol syndrome") OR ("FAS") OR ("social determinants of health") OR ("education*") OR ("social outcome*") OR ("health service*") OR ("justice*") OR ("police") OR ("criminal*") OR ("crime*") OR ("child protection") OR ("welfare*") OR ("mental health service*") OR ("youth service*")) | |
| 2 | (("Child") OR ("Children") OR ("Adolescent") OR ("Adolescents") OR ("Adolescence") OR ("Youth") OR ("Youths") OR ("Teenage*")) | |
| 3 | (((Kimberley) OR ("Bilingurr") OR ("Broome") OR ("Cable Beach") OR ("Camballin") OR ("Dampier Peninsula") OR ("Derby") OR ("Derby-West Kimberley") OR ("Djugun") OR ("Eighty Mile Beach") OR ("Fitzroy Crossing") OR ("Geegully Creek") OR ("Gingerah") OR ("Hall Point") OR ("Halls Creek") OR ("Kimbolton") OR ("King Leopold Ranges") OR ("Koolan") OR ("Kununurra") OR ("Lagrange") OR ("McBeath") OR ("Meda") OR ("Minyirr") OR ("Mount Hardman") OR ("Roebuck") OR ("St George Ranges") OR ("Turkey Creek") OR ("Warmun") OR ("Waterbank") OR ("Willard") OR ("Wyndham") OR ("Wyndham-East Kimberley"))) | |
| 4 | 1 AND 2 | |
| 5 | 4 AND 3 | |
| 6 | Limit 5 to year 1967-current | |
| 7 | Limit 6 to English Language | |
