# Supplementary material for: Health and well-being of children and adolescents living in the Kimberley region of Western Australia: a scoping review protocol
Source: BMJ Open. 2025 Aug 6;15(8):e098268. doi: 10.1136/bmjopen-2024-098268 (PMC12336523; doi:10.1136/bmjopen-2024-098268)
Supplement: Supplementary file 2 [file bmjopen-2024-098268.reviewer_comments.pdf]

PEER REVIEW HISTORY

BMJ Open publishes all reviews undertaken for accepted manuscripts. Reviewers are asked to complete a checklist review form and are provided with free text boxes to elaborate on their assessment. These free text comments are reproduced below.

ARTICLE DETAILS

Title (Provisional)

The Health and Wellbeing of Children and Adolescents Living in the Kimberley Region of Western Australia: A Scoping Review Protocol

Authors

Cannon, Lisa; Carter, Emily; Davies, Jadnah; Thomas, Sue; Elliott, EJ; Rice, Lauren J

VERSION 1 - REVIEW

Reviewer 1

Name Jensen, Todd M.

Affiliation School of Social Work, University of North Carolina at Chapel Hill, Chapel Hill, North Carolina, USA

Date 17-Jan-2025

COI None

I reviewed the protocol entitled, "The Health and Wellbeing of Children and Adolescents Living in the Kimberley Region of Western Australia: A Scoping Review Protocol." I believe the authors should be commended for their focus on this topic. Overall, the protocol rationale is clear and well-articulated. The intended benefits of the conduct of the proposed scoping review also are cogently described. Use of PRISMA-ScR guidelines is a strength. Below I provide several comments intended to support efforts to strengthen the protocol.

1) Consider re-wording the following sentence in the Abstract (particularly use of "reviews" at the end of the sentence): "A second reviewer will independently review 20% of title, abstract, and full-text reviews."

2) What is meant by the following parenthetical statement is not clear: "(West Kimberley is 60.2%)." Consider clarifying.

3) Consider simplifying the following sentence: "The high proportion of Aboriginal people living in the area and their efforts to protect their land and culture have meant that the region remains prosperous in the traditional Aboriginal way of life, with many people still

living in their language and sophisticated kinship systems on or near their land and practising their cultural traditions and customs."

4) Overall, the manuscript would benefit from additional proofreading and editing in an effort to simplify content, address grammatical challenges, and correct typos.

5) Although a definition of "place-based" is provided, it remains unclear what is meant by the conduct of a "place-based scoping review." How would a place-based scoping review differ from a scoping review, methodologically speaking? If the goal is to leverage scoping review findings to shape place-based change efforts, then I am not sure the review approach needs to be labeled "place-based scoping review," as this might confuse readers.

6) It is unclear why the authors have selected 1967 as the earliest year of interest for the scoping review. More rationale should be provided for this choice. The dates of interest for included studies will need to be very carefully selected and justified.

7) It would be helpful if the authors provided more details related to the inclusion and exclusion criteria. For instance, are only empirical articles of interest (articles presenting empirical analyses of quantitative or qualitive data)? Will existing literature reviews, syntheses, or meta-analyses be included? Will theoretical or conceptual articles be included? Based on descriptions related to the data extractions process, the reader might infer that these various article types are relevant. However, it would be helpful to make this explicit when describing inclusion and exclusion criteria.

8) When the authors state that only "peer-reviewed" article types will be included, does this mean that only peer-reviewed articles published in academic journals will be included? If so, why have the authors opted to exclude other types of records, such as doctoral dissertations, master theses, book chapters, etc. (i.e., gray literature)? There might be significant value in including a wide range of record types that fall outside the typical academic publishing process. In the event the authors would like to include gray literature, additional databases might be worth including that specifically target gray literature.

9) When describing the proposed search terms, it is unclear whether the authors intend to use various Boolean operators (e.g., AND, OR). More details should be provided, such that readers could replicate the search process with precision. Also, did the authors consider consulting with a university librarian to help inform the selection of optimal search terms and databases?

10) The use of Covidence is a strength, and I would recommend the authors strongly consider using Covidence for all stages of the review process, including title/abstract screening. Note that Covidence enables the deduplication of records as well. As a result, the authors might consider exporting their search yields from various databases and then uploading those directly to Covidence.

11) More details are needed about how many individuals will participate during the title/abstract screening stage. It is common practice to have at least two screeners

independently screen records (and Covidence can facilitate this very nicely). It also is good practice to have at least two individuals independently review full texts. Having two or more individuals involved in the extraction of study data for each record also can be a strong component of a scoping review. Have the authors considered this? Then, researchers can estimate inter-rater agreement (e.g., agreement percentage, Cohen's kappa) at each major stage of the review process (screening, full-text review, data extraction). As a quick additional note, the data extraction targets proposed appear robust and in alignment with the focal research questions.

**Reviewer**	**2**
**Name**	**Tanaka, Eizaburo**
**Affiliation**	**Hyogo Institute for Traumatic Stress**
**Date**	**13-May-2025**
**COI**	**None**

Please find below my major and minor feedback for this manuscript.

Major Comments

Introduction and Rationale:

The manuscript provides some context (e.g. concerns that research is not informing policy) but could more explicitly articulate the knowledge gap and justify why a scoping review is needed. PRISMA-ScR guidance emphasises explaining the rationale in the context of what is already known. For instance, the introduction could state whether any prior reviews exist (it appears none focuses specifically on the Kimberley) and clarify that a broad scoping approach is appropriate for mapping the range of outcomes and identifying gaps in the literature. Currently, the authors outline the review's purpose but adding a sentence explaining why a scoping methodology (rather than a systematic review or another approach) is suitable would strengthen the rationale.

Eligibility Criteria:

The inclusion criteria are generally appropriate and broad. However, some specific criteria should be further justified or clarified for transparency.

Date Range: The protocol includes studies from January 1967 to the present. As it is uncommon to select 1967 as a start date, the authors should clarify the rationale for this choice.

Peer-Reviewed Literature Only: The protocol restricts inclusion to peer-reviewed literature, excluding grey literature (e.g. government reports and NGO publications). While this is understandable to maintain quality and feasibility, important evidence from local reports or policy documents might be overlooked. The authors should justify this decision and/or acknowledge it as a limitation.

Study Selection Process:
The protocol's study selection method could be more rigorous or transparent. As written, it appears that title/abstract screening will be conducted by "the team", though the number of reviewers involved is unclear, and references marked "unsure" will proceed to full-text review. It is not explicitly stated whether two independent reviewers will screen each citation. The authors should clarify the screening procedure at this stage. Best practice is to have at least two reviewers independently screen titles and abstracts to reduce bias.

Furthermore, full-text screening is to be carried out primarily by one reviewer (LC), with a second reviewer checking only 20% of the full texts for consistency. Discrepancies will be resolved through discussion, or with arbitration by a third reviewer. While this partial double-screening offers a form of quality control, it may not fully mitigate selection bias. Typically, scoping reviews use two independent reviewers for all full texts (or at least a large sample) to ensure no eligible studies are missed. If resources permit, the authors should consider increasing the proportion of double-screened full texts. If only a subset is to be reviewed in duplicate, the authors should explain why 20% was considered sufficient.

Minor Comments
Clarity and Consistency of Terminology:
Ensure consistent usage and formatting of key terms. For example, "Aboriginal" should be capitalised even in compound forms—the table currently says "non-aboriginal children", which should be "non-Aboriginal children" for consistency and respect. Similarly, ensure that "Kimberley" is spelled correctly throughout. In Table 4, it appears as "Kimberly", which should be corrected.

Search Strategy Details:
Overall, the search strategy is comprehensive and multidisciplinary, which is a strength. A couple of minor suggestions:

Please consider adding terms for infants and young children to the search keywords. The current population search terms include “Child*”, “Adolescent*”, “Youth*”, and “Teenage*”, but not terms like “infant”, “newborn”, or “toddler”. Since the review includes ages 0–18, including these terms may help capture studies focusing on the very young.

The list of databases includes MEDLINE via both Ovid and EBSCOhost. It is unusual to search the same database on two platforms. This may reflect institutional access to specific subsets (e.g. Education Source). To avoid confusion, please clarify in the manuscript whether these are distinct searches or if MEDLINE was searched only once. If it was searched on both platforms, note that duplicates will be removed during de-duplication in EndNote.

Risk of Bias Assessment:
The protocol does not currently mention any plan for quality appraisal of included studies, which is perfectly acceptable for a scoping review (as these typically do not assess study quality). However, to avoid any ambiguity, it would be advisable to state this explicitly in the methods section.

Protocol Registration:
Consider adding a note on whether the scoping review protocol has been registered or published elsewhere. PRISMA-ScR item 5 recommends reporting any protocol registration (e.g. with the Open Science Framework). If this protocol has not been registered (which is common, as PROSPERO does not accept scoping reviews), a simple statement such as “This protocol has not been registered” would suffice.

Conclusion
Overall, the protocol is well-structured and addresses most of the criteria outlined in the Arksey and O’Malley framework and PRISMA-ScR checklist. I hope that incorporating the above suggestions will improve its clarity, transparency, and methodological rigour. The topic is important, and the methods are generally sound. Once these revisions are made, the protocol should be in excellent shape for publication.

VERSION 1 - AUTHOR RESPONSE

Reviewer 1: Dr. Todd M. Jensen, School of Social Work, University of North Carolina at Chapel Hill, Chapel Hill, North Carolina, USA

Reviewer comments	Authors Response
I reviewed the protocol entitled, “The Health and Wellbeing of Children and Adolescents Living in the Kimberley Region of Western Australia: A Scoping Review Protocol.” I believe the authors should be commended for their focus on this topic. Overall, the protocol rationale is clear and well-articulated. The intended benefits of the conduct of the proposed scoping review also are cogently described. Use of PRISMA-ScR guidelines is a strength. Below I provide several comments intended to support efforts to strengthen the protocol.	We appreciate your helpful comments and have revised the paper in response.
1) Consider re-wording the following sentence in the Abstract (particularly use of “reviews” at the end of the sentence): “A second reviewer will independently review 20% of title, abstract, and full-text reviews.”	Now reads: “A second reviewer will independently screen 20% of the titles, abstracts, and full-texts.”
2) What is meant by the following parenthetical statement is not clear: “(West Kimberley is 60.2%).” Consider clarifying.	Now reads: “(Aboriginal people represent 60.2% of the population in West Kimberley)”
3) Consider simplifying the following sentence: “The high proportion of Aboriginal people living in the area and their efforts to protect their land and culture have meant that the region remains prosperous in the traditional Aboriginal way of life, with many people still living in their language and sophisticated kinship systems on or near their land and practising their cultural traditions and customs.”	This sentence has been simplified.
4) Overall, the manuscript would benefit from additional proofreading and editing in an effort to simplify content, address grammatical challenges, and correct typos.	We have proofread and edited the manuscript.
5) Although a definition of “place-based” is provided, it remains unclear what is meant by the conduct of a “place-based scoping review.” How would a place-based scoping review differ from a scoping review, methodologically speaking? If the goal is to leverage scoping review findings to shape place-based change efforts, then I am not sure the review approach needs to be labeled “place-based scoping review,” as this might confuse readers.	Our intent was as you described. To remove any confusion, we have removed ‘place-based’ from ‘place-based scoping review’.
6) It is unclear why the authors have selected 1967 as the earliest year of interest for the scoping review. More rationale should be provided for this choice. The dates of interest for included studies will need to be very carefully selected and justified.	To explain why 1967 has been chosen, the below text has now been added under subheading “Eligibility criteria”: “The date of 1967 has been chosen to align with the 1967 referendum, in which 90.77% of Australians voted to amend the Constitution to remove discriminatory references to Aboriginal and Torres Strait Islander peoples.”
7) It would be helpful if the authors provided more details related to the inclusion and exclusion criteria. For instance, are only empirical articles of interest (articles presenting empirical analyses of quantitative or qualitive data)? Will existing literature reviews, syntheses, or meta-analyses be included? Will	The following has been added to the ‘Eligibility criteria’ section: “There are no limits on the type of analyses or study design. Articles presenting qualitative data, quantitative data, meta-analyses as well as literature reviews, theoretical and conceptual articles will be included.”

theoretical or conceptual articles be included? Based on descriptions related to the data extractions process, the reader might infer that these various article types are relevant. However, it would be helpful to make this explicit when describing inclusion and exclusion criteria.	
8) When the authors state that only "peer-reviewed" article types will be included, does this mean that only peer-reviewed articles published in academic journals will be included? If so, why have the authors opted to exclude other types of records, such as doctoral dissertations, master theses, book chapters, etc. (i.e., gray literature)? There might be significant value in including a wide range of record types that fall outside the typical academic publishing process. In the event the authors would like to include gray literature, additional databases might be worth including that specifically target gray literature	When formulating the research question, grey literature was initially considered. We agree it would provide valuable insight. When conducting the initial search of several databases to identify keywords and potential search terms, it became apparent that the database searches will result in a high number of articles. At the time we also conducted a brief search of grey literature, including searching government websites. We realised it would not be feasible to include both types of literature in the same review. This review is restricted to scientific articles. The above is now noted in the manuscript. If funding permits, we will conduct a separate grey literature search.
9) When describing the proposed search terms, it is unclear whether the authors intend to use various Boolean operators (e.g., AND, OR). More details should be provided, such that readers could replicate the search process with precision. Also, did the authors consider consulting with a university librarian to help inform the selection of optimal search terms and databases?	The notes for Table 3 now include: "The Boolean operator 'OR' was included between each search term and 'AND' was used to connect each concept."<br>As stated at the end of the 'Search Strategy' section, "the search strategy for PubMed Central, including limits, is provided in Supplementary Material 1." This search strategy should provide enough information for the reader to replicate the search. The below has been added to the 'search strategy' section: "We consulted with a librarian when formulating the list of search interface/database and the search terms listed in Table 2 and Table 3."
10) The use of Covidence is a strength, and I would recommend the authors strongly consider using Covidence for all stages of the review process, including title/abstract screening. Note that Covidence enables the deduplication of records as well. As a result, the authors might consider exporting their search yields from various databases and then uploading those directly to Covidence	Thank you for your comment. Covidence will be used for everything other than abstract/title as this has already begun. We will consider using Covidence for all stages in future research.
11) More details are needed about how many individuals will participate during the title/abstract screening stage. It is common practice to have at least two screeners independently screen records (and Covidence can facilitate this very nicely). It also is good practice to have at least two individuals independently review full texts. Having two or more individuals involved in the extraction of study data for each record also can be a strong component of a scoping review. Have the authors considered this? Then, researchers can estimate inter-rater agreement (e.g., agreement percentage, Cohen's kappa) at each major stage of the review process (screening, full-text	The 'Study Selection' section and 'Data Extraction' section have been updated to reflect the below.<br><br><u>Title/abstract screening stage:</u> At least two researchers will conduct title/abstract screening, independently. Given the place-based focus of this review, we expect a large portion of excluded papers will be studies that were not conducted in the Kimberley region of Western Australia and thus will not require screening by two researchers. However, to reduce the premature exclusion of potentially relevant papers, we have included an 'unsure' option, allowing researchers to flag articles

review, data extraction). As a quick additional note, the data extraction targets proposed appear robust and in alignment with the focal research questions.	they are not confident in including/excluding. These articles will be reviewed by two researchers who will meet and discuss.<br><br>Full-text screening stage: As we anticipate many publications will result from our search, a second researcher will initially conduct a full-text review for 20% of papers, and we will then decide how many full-texts they will review, depending on the level of agreement between reviewers.<br><br>Data extraction: Thank you for your comments about a second reviewer for the data extraction. This was carefully considered. Given the large number of articles anticipated, we plan to use the same method as used in the abstract and full-text screening. There is an 'additional notes' section on the data extraction template and the reviewers will be asked to flag if they feel data extraction was ambiguous in anyway and a second reviewer will then extract data for that article. When writing up the findings, authors will review full text to ensure the final summary accurately reflects the original publication.

Reviewer 2: Dr. Eizaburo Tanaka, Hyogo Institute for Traumatic Stress

Reviewer comments	Authors Response
**1) Introduction and Rationale:**<br>The manuscript provides some context (e.g. concerns that research is not informing policy) but could more explicitly articulate the knowledge gap and justify why a scoping review is needed. PRISMA-ScR guidance emphasises explaining the rationale in the context of what is already known. For instance, the introduction could state whether any prior reviews exist (it appears none focuses specifically on the Kimberley) and clarify that a broad scoping approach is appropriate for mapping the range of outcomes and identifying gaps in the literature. Currently, the authors outline the review's purpose but adding a sentence explaining why a scoping methodology (rather than a systematic review or another approach) is suitable would strengthen the rationale.	Thank you for your feedback. We now note in the final paragraph of introduction that no prior reviews have been conducted on this topic. This paragraph explicitly outlines why we are conducting this review and what benefit it would provide.<br><br>Sentence added to 'protocol design' section: "Given our aim is to provide a broad overview of the literature, we have chosen a scoping review rather than a systematic review."
**2) Eligibility Criteria:**<br>The inclusion criteria are generally appropriate and broad. However, some specific criteria should be further justified or clarified for transparency.	More details have been added to the 'Eligibility criteria' section.
**3) Date Range:** The protocol includes studies from January 1967 to the present. As it is uncommon to select 1967 as a start date, the authors should clarify the rationale for this choice.	To explain why 1967 has been chosen, the below text has now been added under subheading "Eligibility criteria": "The date of 1967 has been chosen to align with the 1967 referendum, in which 90.77% of Australians voted to amend the Constitution to

	remove discriminatory references to Aboriginal and Torres Strait Islander peoples.”
**4) Peer-Reviewed Literature Only:** The protocol restricts inclusion to peer-reviewed literature, excluding grey literature (e.g. government reports and NGO publications). While this is understandable to maintain quality and feasibility, important evidence from local reports or policy documents might be overlooked. The authors should justify this decision and/or acknowledge it as a limitation.	When formulating the research question, grey literature was initially considered. We agree it would provide valuable insight. When conducting the initial search of several databases to identify keywords and potential search terms, it became apparent that the database searches will result in a high number of results. At the time we also conducted a brief search of grey literature, including searching a few government websites. We realised it would not be feasible to include both types of literature in the same review. This review is restricted to scientific articles. The above is now noted in the manuscript. If funding permits, we will conduct a separate grey literature search.
**5) Study Selection Process:**<br>The protocol’s study selection method could be more rigorous or transparent. As written, it appears that title/abstract screening will be conducted by “the team”, though the number of reviewers involved is unclear, and references marked “unsure” will proceed to full-text review. It is not explicitly stated whether two independent reviewers will screen each citation. The authors should clarify the screening procedure at this stage. Best practice is to have at least two reviewers independently screen titles and abstracts to reduce bias.<br><br>Furthermore, full-text screening is to be carried out primarily by one reviewer (LC), with a second reviewer checking only 20% of the full texts for consistency. Discrepancies will be resolved through discussion, or with arbitration by a third reviewer. While this partial double-screening offers a form of quality control, it may not fully mitigate selection bias. Typically, scoping reviews use two independent reviewers for all full texts (or at least a large sample) to ensure no eligible studies are missed. If resources permit, the authors should consider increasing the proportion of double-screened full texts. If only a subset is to be reviewed in duplicate, the authors should explain why 20% was considered sufficient.	The ‘Study Selection’ section and ‘Data Extraction’ section have been updated to reflect the below.<br><br><u>Title/abstract screening stage:</u> At least two researchers will conduct title/abstract screening, independently. Given the place-based focus of this review, we expect a large portion of excluded papers will be studies that were not conducted in the Kimberley region of Western Australia and thus will not require screening by two researchers. However, to reduce the premature exclusion of potentially relevant papers, we have included an ‘unsure’ option, allowing researchers to flag articles they are not confident in including/excluding. These articles will be reviewed by two researchers who will meet and discuss.<br><br><u>Full-text screening stage</u>: As we anticipate many publications will result from our search, a second researcher will initially conduct a full-text review for 20% of papers, and we will then decide how many full-texts they will review, depending on the level of agreement between reviewers.
**6) Clarity and Consistency of Terminology:**<br>Ensure consistent usage and formatting of key terms. For example, “Aboriginal” should be capitalised even in compound forms—the table currently says “non-aboriginal children”, which should be “non-Aboriginal children” for consistency and respect. Similarly, ensure that “Kimberley” is spelled correctly throughout. In Table 4, it appears as “Kimberly”, which should be corrected.	Thank you for noticing these spelling mistakes. We have corrected these and proofread the manuscript.
**7) Search Strategy Details:**	Thank you, the additional terms and removal of Medline from one interface would have been

Overall, the search strategy is comprehensive and multidisciplinary, which is a strength.<br>A couple of minor suggestions:<br>Please consider adding terms for infants and young children to the search keywords. The current population search terms include "Child*", "Adolescent*", "Youth*", and "Teenage*", but not terms like "infant", "newborn", or "toddler". Since the review includes ages 0–18, including these terms may help capture studies focusing on the very young.<br>The list of databases includes MEDLINE via both Ovid and EBSCOhost. It is unusual to search the same database on two platforms. This may reflect institutional access to specific subsets (e.g. Education Source). To avoid confusion, please clarify in the manuscript whether these are distinct searches or if MEDLINE was searched only once. If it was searched on both platforms, note that duplicates will be removed during deduplication in EndNote.	appropriate. The search is already underway, but we will consider this in future research. The duplicates will be removed during the deduplication in EndNote as mentioned under the 'Study Selection' section.
**8) Risk of Bias Assessment:**<br>The protocol does not currently mention any plan for quality appraisal of included studies, which is perfectly acceptable for a scoping review (as these typically do not assess study quality). However, to avoid any ambiguity, it would be advisable to state this explicitly in the methods section.	The aim of the review is to provide a broad overview of outcomes of children and adolescents, with the focus on the recommendations provided in the papers. As it is the recommendations that will help inform improvement in policy and practice. The 'Data extraction' section now includes: "The focus of this review is to identify broad research outcome topics and recommendations rather than examine the quality of specific studies. We therefore will not conduct risk of bias assessments."
**9) Protocol Registration:**<br>Consider adding a note on whether the scoping review protocol has been registered or published elsewhere. PRISMA-ScR item 5 recommends reporting any protocol registration (e.g. with the Open Science Framework). If this protocol has not been registered (which is common, as PROSPERO does not accept scoping reviews), a simple statement such as "This protocol has not been registered" would suffice.	We had looked into registering the review in PROSPERO, but as you know they do not accept scoping reviews.
**10)Conclusion**<br>Overall, the protocol is well-structured and addresses most of the criteria outlined in the Arksey and O'Malley framework and PRISMA-ScR checklist. I hope that incorporating the above suggestions will improve its clarity, transparency, and methodological rigour. The topic is important, and the methods are generally sound. Once these revisions are made, the protocol should be in excellent shape for publication.	We appreciate your helpful comments and have revised the paper in response.

VERSION 2 - REVIEW

Reviewer 1

Name Jensen, Todd M.

Affiliation School of Social Work, University of North Carolina at Chapel Hill, Chapel Hill, North Carolina, USA

Date 23-Jun-2025

COI

I believe the authors have been highly responsive to each of my comments. I am satisfied with the responses (and resultant revisions when applicable) provided, and I have no further comments at this stage.

Reviewer 2

Name Tanaka, Eizaburo

Affiliation Hyogo Institute for Traumatic Stress

Date 17-Jun-2025

COI

Thank you for your updated manuscript. I have no additional comments.
